# Enduring and Stable Surface Dielectric Barrier Discharge (SDBD) Plasma Using Fluorinated Multi-Layered Polyimide

**DOI:** 10.3390/polym10060606

**Published:** 2018-06-02

**Authors:** Dongliang Bian, Yun Wu

**Affiliations:** 1 Science and Technology on Plasma Dynamics Laboratory, Air Force Engineering University, Xi’an 710038, China; biandl1990@163.com (D.B.); 2 Institute of Aero-engine, School of Mechanical Engineering, Xi’an Jiaotong University, Xi’an 710049, China

**Keywords:** surface fluorination, SDBD, discharge characteristic, multi-layered polyimide

## Abstract

In this work, multi-layered polyimide (PI) films were surface fluorinated at 328 K and 0.05 MPa using F_2_/N_2_ mixture with 20% F_2_ by volume, for a fluorination time of 0, 30 and 60 min, respectively. Then, they were subjected to discharge plasma as barrier dielectrics of surface dielectric barrier discharge (SDBD) at ambient atmospheric air. The dielectric lifetime of SDBD greatly extends after 60 min surface fluorination. In addition, optical emission spectroscopy (OES) results indicate that during the plasma processing, SDBD with fluorinated PI can obtain more stable plasma parameters, including gas temperature and electron temperature. Dielectric surface properties were further evaluated by infrared thermography, scanning electron microscope (SEM), and X-ray photoelectron spectroscopy (XPS). It is considered that both physical and chemical effects lead to the extension of dielectric lifetime. The physical effect is reflected in low surface temperature and increased surface roughness, while the chemical effect is reflected in the graft of fluorine groups.

## 1. Introduction

Nowadays, atmospheric plasma sources have drawn more attention due to their many advantages, such as having no need for vacuum equipment, being a low-cost and simple system, and being easy to be operated [1]. Among various types of plasma sources, surface dielectric barrier discharge (SDBD) has attracted significant attention because it can produce stable discharge with very simple configuration. Thus, it has been characterized [2,3] and successfully applied as a plasma actuator in various aerodynamic fields, such as active flow control [2,4], heat generation for de-icing and anti-icing [5], as well as film cooling [6]. Besides, it can be used as a plasma-based chemical reactor in many environmental applications, such as ozone generation [7,8], decomposition of volatile organic compounds (VOCs) [9], NO_x_ conversion [10], and inactivation of killing bacteria [11]. Generally, SDBD plasma device is made of a dielectric plate with two metallic electrodes placed on each side. Supplied with an AC voltage or a pulsed voltage, non-thermal plasma is created on the surface of dielectric. Typical barrier dielectric materials include ceramics, polymers, quartz, and glass. 

Compared with traditional ceramics or glasses, polymers possess the advantage of flexibility so that they can be easily adhered to the curved surface, such as the leading edge of the wings, aero engine blades, and many cylindrical-shaped plasma reactors. However, it has been proved that polymers have poor resistance to the plasma environment; the intense bombardment of energetic active species (ions, electrons, UV photons, etc.) can erode them [12,13]. As a result, lack of durability becomes a serious drawback for polymer-based SDBD plasma devices. Pons et al. [12] showed that degradation of two sorts of polymers (Poly methyl methacrylate and Polyvinyl chloride) occurred when they were exposed to 10 min of plasma operation in ambient air. Hanson et al. [14] and Ndong et al. [15] experimentally showed that degradation of PI-based actuators was accompanied by an increase of power consumption after plasma processing. Rigit et al. [16] found that plasma actuators manufactured from PCBs were visually shown to degrade and fail in some instances.

From the above reports, it can be concluded that appropriate pretreatment of the polymers needs to be done to obtain better performance of SDBD plasma devices. As one of the most effective approaches to the chemical modification of polymer surfaces, direct fluorination using fluorine gas has been widely developed from fundamental research to industrial applications [17]. It has advantages such as outstanding surface properties, similar to the fluoropolymers that can be obtained without changing the bulk characteristics of the starting polymer. The research works and applications are mainly concentrated on the improvements of the barrier properties, wettability, adhesiveness, chemical stability or biocompatibility of polymers [18,19] and dealing with the electrical properties of polymers. For example, An et al. [20,21] indicated that surface fluorination of PE has a significant influence on the charge injection and accumulation. Du et al. [22,23] found that surface fluorination is an effective method for modulating the electrical properties and restraining the surface charge and space charge accumulation of the PI film.

In recent years, several groups have modified polymers’ surface chemical composition with the help of plasma in order to increase their adhesion or hydrophilicity [24,25,26,27]. During plasma processing, ion bombardment breaks chemical bonds in the polymer’s backbones, while radicals enable functional groups to be grafted onto the polymer’s surface. The functional groups improve the polymer’s wettability and adhesion by inducing an increase of its surface energy. Similar to plasma surface modification, when a SDBD plasma device is powered, the produced active species in the discharge region also interact with the dielectric surface. Thus, the existing research methods of plasma surface modification may provide a new route to investigate the degradation issue of polymer-based plasma devices. In our previous work [28], PI/Al_2_O_3_ nanocomposite was fabricated and used as a dielectric of the plasma actuator. Compared to the conventional PI-based actuator, both the dielectric lifetime was enhanced and the mechanical performance was improved. The major purpose of the present investigation is to understand the effects of varying fluorination time on the characteristics of PI-based SDBD plasma devices. Firstly, discharge properties including power consumption and dielectric capacitance were evaluated. Then, dielectric lifetimes, OES, and surface temperature distribution was measured and compared. Complementarily, in-depth studies of surface morphology and chemical composition by the way of SEM and XPS were conducted to characterize the surface properties of the dielectrics.

## 2. SDBD Device and Experimental Set up

### 2.1. SDBD Device

Five-layer PI films were chosen as dielectric barriers in this work, bonded with silicone adhesive. The PI film had a thickness of 50 µm and an area of 50 × 50 mm^2^. A 30 µm thickness silicon adhesive was obtained from 3M Co. Ltd., Hongkong, China. The total thickness of the barrier dielectric was 0.37 mm. Surface fluorination of the dielectrics was carried out in a stainless reactor kettle with polytetrafluoroethylene lining at about 328 K (55 °C) using a F_2_/N_2_ mixture with 20% F_2_ by volume [22]. After evacuation and purification with nitrogen gas more than three times, the reactive gas mixture was introduced slowly into the vessel until the mixture pressure in the vessel had reached 0.05 MPa (500 mbar). The fluorination time was 30 and 60 min, respectively. After treatment, the reactive gas mixture was purged from the vessel with nitrogen. 

**Figure 1:**
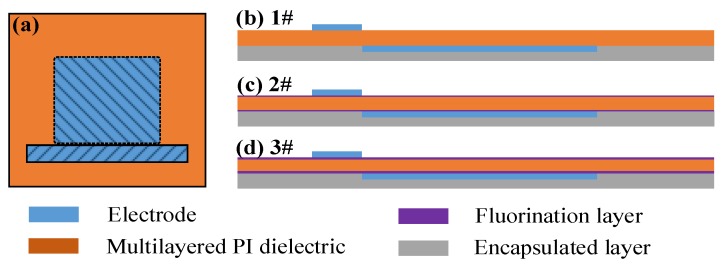
Top view of Surface Dielectric Barrier Discharge (SDBD) plasma device (**a**) and cross section of SDBD plasma devices with un-fluorinated PI (**b**), 30 min fluorinated PI (**c**) and 60 min fluorinated PI (**d**); Not scale.

Once the fluorination process was completed, electrodes were fabricated using copper tapes with a thickness of 70 µm, as shown in Figure 1a. The width of the exposed and encapsulated electrode was 5 and 25 mm, respectively. The tips of the encapsulated electrode were rounded with a diameter of 0.8 mm. There was no gap between the two edges of the electrodes. Their effective span length (along which plasma was generated) was 30 mm. The encapsulated electrodes were covered in Kapton tape to prevent unwanted plasma formation. As shown in Figure 1b–d, the top device (1#) was based on the un-fluorinated PI. The middle device (2#) was based on 30 min fluorinated PI. The bottom device (3#) was based on 60 min fluorinated PI.

### 2.2. Experimental Setup

**Figure 2:**
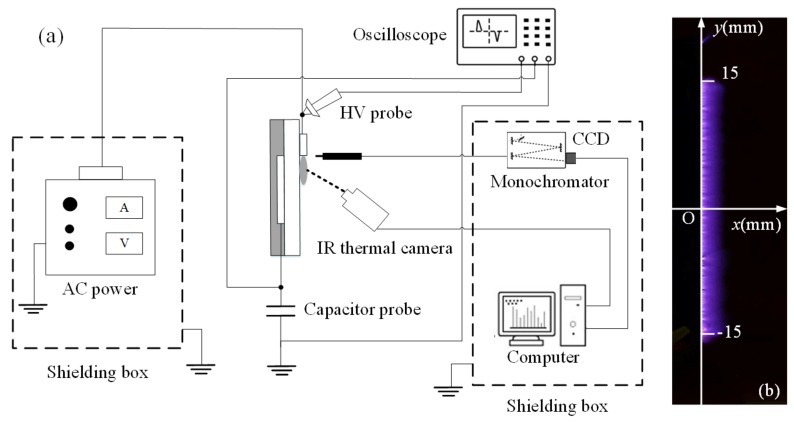
Schematic diagram of the experimental setup (**a**) and top view of discharge plasma (**b**).

The experimental setup is schematically illustrated in Figure 2a, which is composed of an AC power supply, a SDBD plasma device, an electrical measurement system, and optical and thermal detection systems. The AC power (CTP-2000K, Suman Co., Nanjing, China) can supply an adjustable high sine peak to peak voltage (*V*_pp_) up to 30 kV, with a power frequency (*f*) in a range of 4–18 kHz. Surface plasma was generated near the exposed electrode. The photographs were captured using a Nikon D7000 digital camera (Tokyo, Japan). The applied voltage was measured by using a high voltage probe (Tektronix P6015A, Beaverton, OR, USA). Traces of the voltage across the plasma device and the capacitor probe were displayed and recorded using an oscilloscope (DPO4014, Tektronix Co., Beaverton, OR, USA). In order to reduce the interferences of discharge pulses on the detection system and other instruments, the power supply was placed in a shielding box, which was connected to the ground. Figure 2b shows the top view of the surface plasma, where “*x*” is a stream-wise direction (i.e., parallel to a streamer channel), and “*y*” is a span-wise direction (i.e., parallel to the exposed electrode).

Infrared radiation characterization was conducted by measuring the surface temperature using a thermal camera Systems (SC7000, FLIR Inc., Portland, OR, USA) in the spectral range of 7.7–9.3 µm, as was earlier described in Refs. [29,30]. The camera was placed 20 cm in front of the discharge. For each case, the device was run for a duration of 120 s and then switched off for 30 s. The thermal camera and the computer then recorded the distribution of the surface temperature. Before the test, the surface emissivity was determined experimentally by simultaneously recording the temperature measured through the thermal camera and a thermocouple. In this case, the discharge was turned off and the dielectric was heated by an external source. Emissivity was found to be ε = 0.83 ± 0.02 when temperature ranged from 15 to 80 °C. OES was exploited to investigate excited active species generated in the SDBD plasma devices. The optical emission spectra were collected by a charge-coupled device spectrometer (Avantes 2048, Apeldoorn, Netherlands) through an optical fiber, located 5 mm above the surface of the dielectric layer, perpendicular to the plasma devices, and fixed in front of the generated plasma. The integration time of spectrograph was set to be 2000 ms. Optical emission experiments were performed in a dark room and the background noise was measured before each test and subtracted before post-processing. As shown in Figure 2b, each condition was measured 30 times, at the scope *x* = 0 mm and *y* = −15–15 mm, the emission intensities were averaged.

Following the sputter coating of the films with gold, the surface morphologies of the dielectrics were observed using a scanning electron microscope (SEM, Zeiss Supra55, Oberkochen, Germany). X–ray photoelectron spectroscopy (Bestec, Berlin, Germany) employing a monochromatic Al K radiation (1486.6 eV) operated at 5 kV and 10 mA in 10^−10^ mbar was used to determine the surface chemical structures of the dielectrics.

### 2.3. Power Consumption and Dielectric Capacitance Calculation

Measurements of the power consumption were conducted using a capacitor probe for the purpose of integrating the charge-voltage (*Q-V*) cyclograms (or Lissajous figures), which has been elaborated in Refs. [4,14,31]. As shown in Figure 2, a ceramic capacitor probe with capacitance (*C*_pc_) of 10 nF was placed between the encapsulated electrode and the ground. The voltage across the capacitor probe (*V*_pc_) can be measured directly through an oscilloscope. The instantaneous charge, *Q*(*t*), was the product of *V*_pc_(*t*) and *C*_pc_. The input voltage and charge values were plotted against each other to form a closed curve. The area equals to the consumed discharge energy per cycle *E*_k_, which can be calculated by:

(1) $$E_{k}=\oint_{k}C_{\mathrm{pc}}V_{\mathrm{pc}}(t)dV=\oint_{k}Q(t)dV$$

The consumed power *P* was the product of *E*_k_ and *f*:

(2) $$P=Ef=\frac{f}{K}\sum_{k=1}^{k}E_{k}$$

**Figure 3:**
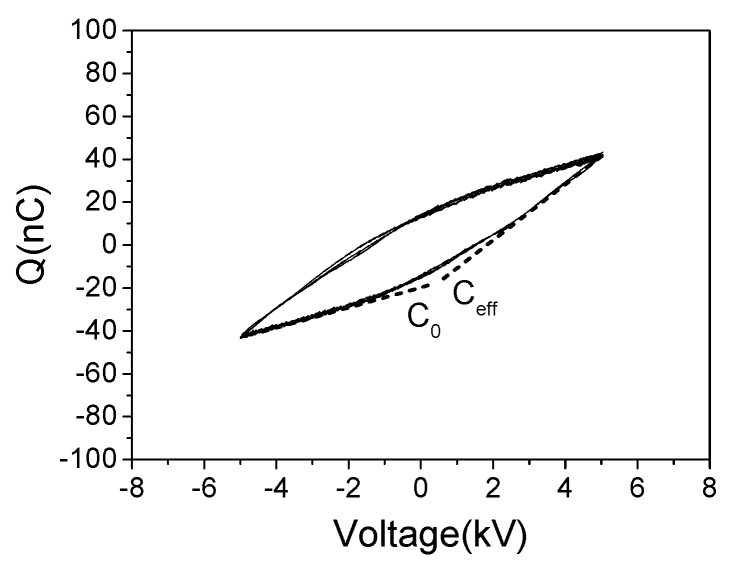
Lissajous figure of the un-fluorinated device (*V*_pp_ = 10 kV, *f* = 6 kHz).

In this work, 100 cycles of Lissajous figure data were calculated to obtain the averaged *E*_k_; the standard deviation was ≤3% for all cases. The consumed power was further calculated through the product of *f* and the averaged *E*_k_. Additionally, Lissajous figure can also be utilized to analyze the discharge capacitance of the PI-based device [31]. As shown in Figure 3, the capacitance was equal to d*Q*/d*V*. Two typical capacitance values were the cold capacitance (*C*_0_) and the effective capacitance (*C*_eff_), which corresponded to the cases when plasma was absent and present, respectively. The calculation method of *C*_0_ and *C*_eff_ was based on a least squares fit of a straight line over the constant-slope regions, more details have been described in Ref. [14]. The uncertainty of the calculated capacitances was less than 3%. It should be pointed out that *C*_0_ value of the un-fluorinated device can also be measured through a multimeter, and the result agreed well with this method. According to Lissajous figure data, the calculated *C*_0_ and *C*_eff_ were 4.5 ± 0.1 and 13.5 ± 0.3 pF, which were three orders and two orders lower than the value of *C*_pc_, respectively. Thus, on the basis of the selection criteria proposed by Kriegseis [31], the capacitance probe was sufficient large and feasible in present work.

## 3. Results and Discussion

### 3.1. Discharge Properties

**Figure 4:**
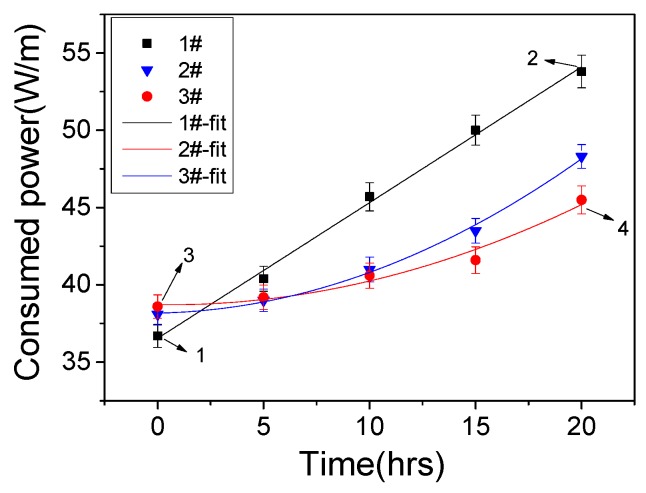
Consumed power of the three plasma devices as a function of the aging time.

Figure 4 shows the time dependence of the consumed power for three plasma devices. It can be seen that the consumed power values of the two fluorinated devices were higher than that of the un-fluorinated one at the aging time of 0 h, then, the consumed power of the three devices increased with the aging time; this was mainly because the plasma was situated nearer the grounded electrode with the degradation of the top PI layer, and therefore in a region where the expected electric field strength was higher [14]. Besides, the power data in case of 1# device can be well fit by a simple linear Function (3), the calculated *A* and *B* values were 0.88 and 36.54, respectively. A second-order polynomial Function (4) can be used to fit the cases of 2# and 3# devices, the calculated *C*, *D* and *E* values were 0.024, 0.027, 38.17 for 2# device and 0.017, −0.017, 38.72 for 3# device. The fitted results show that the plasma processing had less effects on the consumed power values of fluorinated devices. For instance, after aging time of 20 h, the consumed power of 3# device increased by about 18%, which was about 46% lower than the case of 1# device. In Ref. [14], a method using a coating of Polydimethylsiloxane oil was proposed to prevent erosion of the PI-based actuator. Similar phenomenon of slower increase rate of the consumed power has also been observed.

(3) f(x)=Ax+B

where *A* and *B* are free parameters.

(4) $$f(x)=Cx^{2}+Dx+E$$

where *C*, *D* and *E* are free parameters.

**Figure 5:**
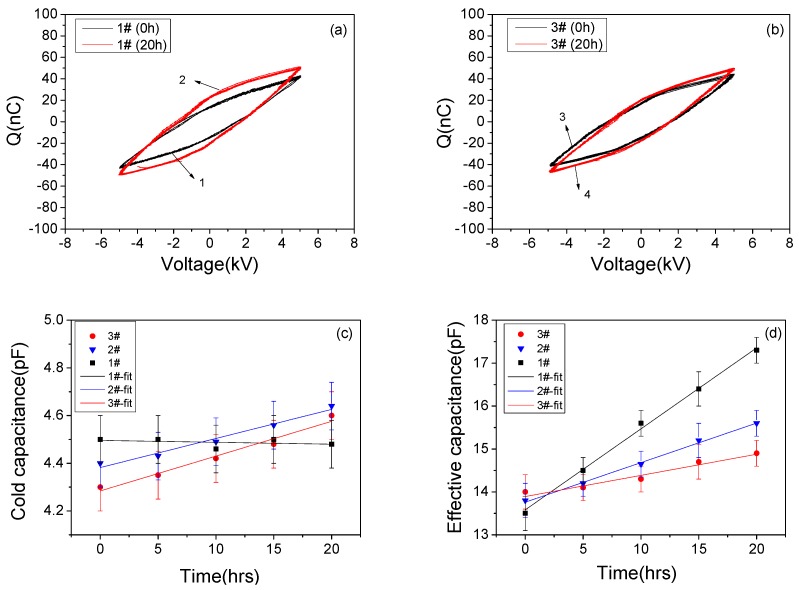
Lissajous figures of un-fluorinated device (**a**) and 60 min fluorinated device (**b**) at the aging times of 0 and 20 h, cold capacitance (**c**) and effective capacitance (**d**) as a function of the aging time for the three devices.

Lissajous figures corresponding to four different discharge cases marked in Figure 4 are shown in Figure 5a,b, for 1# device and 3# device, respectively. It is seen that the shapes of Lissajous figures did not change apparently. However, the enclosed area was expanded for both cases after aging and the case of 1# device seemed more pronounced, which led to higher *E*_k_ and *P*. The calculating results of *C*_0_ and *C*_eff_ values at different aging times for the three devices are given in Figure 5c,d, along with the fits based on Function (3). In case of *C*_0_ fitting, the values of *A* were −8 × 10^−4^, 0.012 and 0.015, while the values of *B* were 4.50, 4.38 and 4.28, for the cases of 1#, 2# and 3# devices, respectively. In case of *C*_eff_ fitting, the values of *A* were 0.188, 0.098 and 0.049; while the values of *B* were 13.58, 13.75 and 13.89, for the cases of 1#, 2# and 3# devices, respectively. Based on the fitted lines, it can be seen that for 1# device, *C*_0_ values remained almost unchanged with the aging time, while *C*_eff_ increased significantly from 13.5 ± 0.3 to 17.3 ± 0.3 pF (about 28% higher). Compared to 1# device, both *C*_0_ and *C*_eff_ values had slight increase for 2# and 3# devices. Taking 3# device as an example, after 20 h of plasma discharge aging, *C*_0_ values increased from 4.3 ± 0.1 to 4.6 ± 0.1 pF, while *C*_eff_ values increased from 14 ± 0.3 to 14.9 ± 0.3 pF (about 6.4% higher), which was much lower than variation range of 1# device. According to Ref. [14], the increase range of *C*_eff_ value directly revealed the degradation degree of the dielectric beneath discharge plasma; it can be concluded that when using fluorinated PI as a dielectric, the dielectric degradation was effectively inhibited.

### 3.2. Dielectric Lifetime

**Figure 6:**
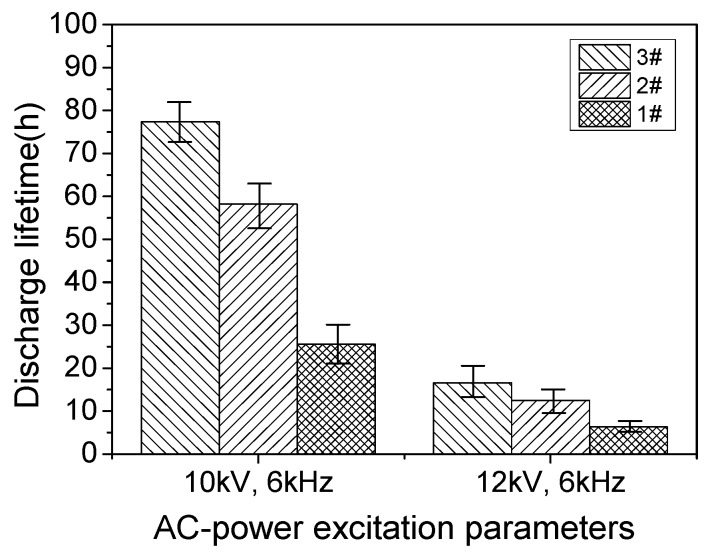
Dielectric lifetime of SDBD plasma devices with different dielectrics under the conditions of the two power supply parameters.

The results of lifetime measurements for PI dielectric with different fluorination time are shown in Figure 6. Two different applied voltages with the same frequency were set as the aging condition: 10 kV, 6 kHz and 12 kV, 6 kHz. For each condition, five samples were tested to obtain the average lifetime. As can be observed, for the same power supply parameter, longer fluorination time brought a gradual longer dielectric lifetime of the PI dielectric. At the applied voltages of 10 and 12 kV, dielectric lifetimes of the 60 min fluorinated PI were around 77 and 16.6 h, about 3 and 2.6 times longer than the case of the un-fluorinated PI. Dielectric lifetimes of the 30 min fluorinated PI were about 58.2 and 12.5 h, about 2.3 and 2 times longer than the case of the un-fluorinated PI. Also, for all these three dielectrics, it can be seen that dielectric lifetimes decreased obviously when the applied voltage was increased from 10 to 12 kV.

### 3.3. Surface Temperature and Plasma Diagnosis

**Figure 7:**
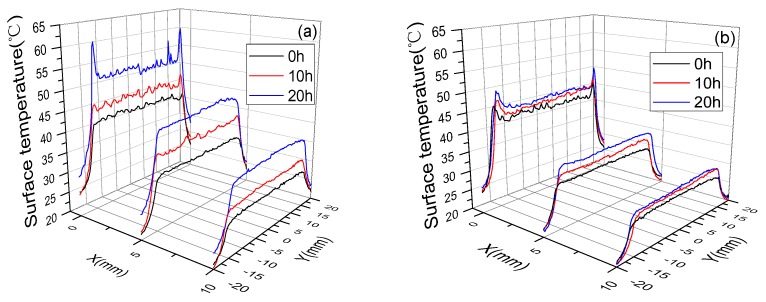

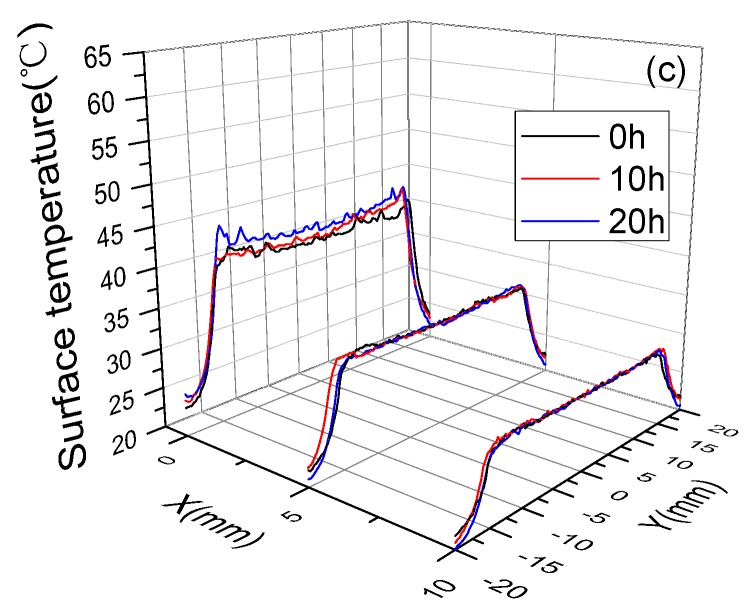
Surface temperature of the dielectrics with 0 min (**a**), 30 min (**b**) and 60 min (**c**) surface fluorination, along the *Z*-direction at different *x* position as a function of the aging time (*V*_pp_ = 10 kV, *f* = 6 kHz).

Surface temperature of three devices at different aging times (0, 10 and 20 h) was measured using a thermal infrared camera. Figure 7 quantitatively gives the temperature distribution along the edge of the exposed electrode (*x* = 0, 5 and 10 mm). For the three devices at different aging times, the *y* direction distribution indicated a maximum temperature rise on the edge of the exposed electrode (*x* = 0 mm). A gradual drop in temperature was observed on the downstream side of the electrode (*x* = 5 and 10 mm). Also, the temperature perturbation at *x* = 0 mm was strongest and the more uniform distribution appeared at a downstream location (*x* = 5 and 10 mm). It is further noted that the surface temperature value in the same positon gradually increased with the aging time, and the surface temperature variations of the un-fluorinated PI were more pronounced and affected by long plasma processing. For instance, after 20 h of discharge aging, the maximum temperature rise at the position of *x* = 0 mm was higher in the case of the un-fluorinated PI (about 19 °C) than the cases of the 30 and 60 min fluorinated PI dielectrics (about 3.6 and 2.9 °C, respectively). At the position of *x* = 20 mm, the maximum temperature of the un-fluorinated PI increases about 9 °C, while no obvious variation shown in Figure 7c can be observed for the 60 min fluorinated PI. 

**Figure 8:**
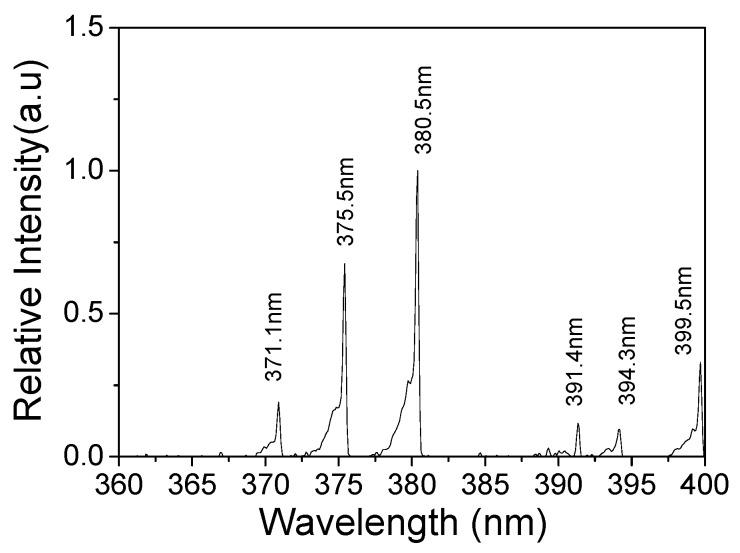
Normalized optical emission spectra of the plasma device with un-fluorinated PI (*V*_pp_ = 10 kV, *f* = 6 kHz).

Optical emission spectroscopy (OES) is used to study discharge plasma. The emission spectrum in the range of 360–400 nm emitted from the plasma device with un-fluorinated PI is shown in Figure 8. The emission intensity was normalized with the intensity at 380.5 nm. As expected with other atmospheric air non-equilibrium discharge [32], N_2_ peaks were dominant in the emission spectra. The major spectra came from the second positive system (SPS) of N_2_ (C^3^П_u_→B^3^П_g_) and the first negative system (FNS) of N_2_^+^ (B^2^Σ_u_^+^→X^2^Σ_g_^+^). Typical peaks were identified according to Refs. [33,34,35], which included N_2_ (C^3^П_u_→B^3^П_g_, 2–4) at 371.1 nm, N_2_ (C^3^П_u_→B^3^П_g_, 1–3) at 375.5 nm, N_2_ (C^3^П_u_→B^3^П_g_, 0–2) at 380.5 nm, N_2_^+^ (B^2^Σ_u_^+^→X^2^Σ_g_^+^, 0–0) at 391.4 nm, N_2_ (C^3^П_u_→B^3^П_g_, 2–5) at 394.3 nm, and N_2_ (C^3^П_u_→B^3^П_g_, 1–4) at 399.5 nm.

**Figure 9:**
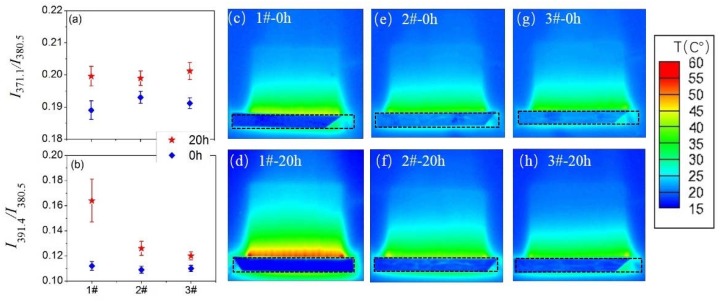
Variation of *I*_371.1_/*I*_380.5_ (**a**), *I*_391.4_/*I*_380.5_ (**b**), surface temperature distributions of 1# device at the aging times of 0 h (**c**) and 20 h (**d**), 2# device at the aging times of 0 h (**e**) and 20 h (**f**), 3# device at the aging times of 0 h (**g**) and 20 h (h).

The typical parameters such as N_2_ (C^3^П_u_) vibrational temperature (*T*_vib_), electron temperature (*T*_e_) and N_2_ (C^3^П_u_) rotational temperature (*T*_rot_) are important parameters to evaluate plasma characteristics. The relative intensity ratio between 371.1 and 380.5 nm (*I*_371.1_/*I*_380.5_) can be used as an indicator of *T*_vib_; the relative intensity ratio between 391.4 and 380.5 nm (*I*_391.4_/*I*_380.5_) is used to obtain the temporal and spatial averaged *T*_e_ by the line-ratio technique of OES [36,37]. The variations of the two parameters at the discharge aging time of 0 and 20 h are shown in Figure 9a,b, respectively. For the three devices at the aging time of 0 h, the *I*_371.1_/*I*_380.5_ and *I*_391.4_/*I*_380.5_ values remained almost the same (around 0.19 and 0.11, respectively), suggesting that there were no conspicuous effects of surface fluorination on *T*_vib_ and *T*_e_. When the discharge time increased to 20 h, *I*_371.1_/*I*_380.5_ values of all devices shown in Figure 9a still had little changes (from around 0.19 to 0.20) during the plasma processing, suggesting that *T*_vib_ had minor dependence on the discharge aging time. Nevertheless, for 1# device shown in Figure 9b, the *I*_391.4_/*I*_380.5_ value increased apparently from 0.11 (0 h) to 0.164 (20 h), indicating a remarkable change of plasma characteristics—an increase in the number of high energy electrons and the average electron energy [38]. Compared to 1# device, the changes of the *I*_391.4_/*I*_380.5_ values for 2# and 3# devices were not obvious (from about 0.11 to 0.12). The different changes of *I*_391.4_/*I*_380.5_ values for three devices can be explained by the distribution of surface temperature. As shown in Figure 9c, at the aging time of 0 h, the temperature distribution was uniform along the edge of the exposed electrode. However, at the aging time of 20 h, intense temperature perturbation can be clearly seen from Figure 9d, which was caused by the severe dielectric degradation of the un-fluorinated PI. As a result, the electrical field distortion became worse and more direct heat injected from the irregular glow spots to the dielectric surface, where the intense concentration of the ionization events occurred randomly [29]. The existence of glow spots in the negative-going cycle meant that the electrons transferred from the exposed electrode to the dielectric surface, combined with the fact that more discharge power was consumed by 1# device after 20 h. It can be inferred that the discharge energy deposited on each electron transport channel also became higher. Therefore, *T*_e_ increased greatly because it was determined by the optical emission from the strong and bright discharge channels. At the same time, no noteworthy temperature perturbations can be seen in Figure 9f,h.

**Figure 10:**
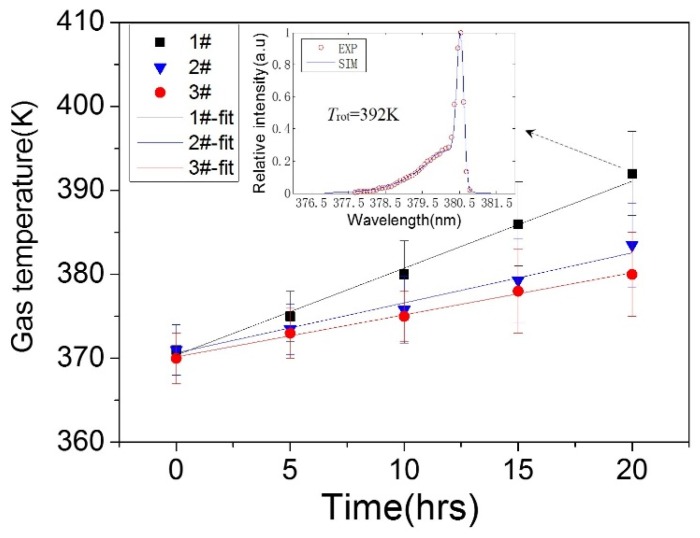
Effects of plasma discharge aging on the gas temperatures of the three devices.

The values of *T*_rot_ are further determined by comparing the experimentally measured data to the theoretically calculated data with a least-square procedure. *T*_rot_ is obtained by making the squared difference between measured and calculated normalized intensity minimum. In addition, gas temperature (*T*_gas_) can be regarded as being almost equal to *T*_rot_ because the equilibrium between rotational and translational motion is easily obtained due to frequent and fierce collisions among the heavy particles [39]. The calculating results of three cases are shown in Figure 10, along with fits based on Function (3). The fitting values of *A* were 1.04, 0.60 and 0.50, while the values of *B* were 370.40, 370.65 and 370.17, for the cases of 1#, 2# and 3# devices, respectively. As presented in the embedded figure, the simulated spectrum at *T*_rot_ = 392 K fits the experimental one with good agreement. It can be seen that plasma discharge aging had more effects on 1# device than the other two devices. After 20 h, the gas temperature of 1# device increased from about 370 to 390 K; this was due to the increase of electron density and electron mean energy, more frequent collisions between electrons and heavy particles occurred, and therefore more energy transferred from electrons to heavy particles. At the same time, the increase ranges of 2# and 3# devices were lower than that of 1# device, suggesting that more stable plasma discharge can be obtained after dielectric surface fluorination.

### 3.4. Surface Morphology and Chemical Structure

**Figure 11:**
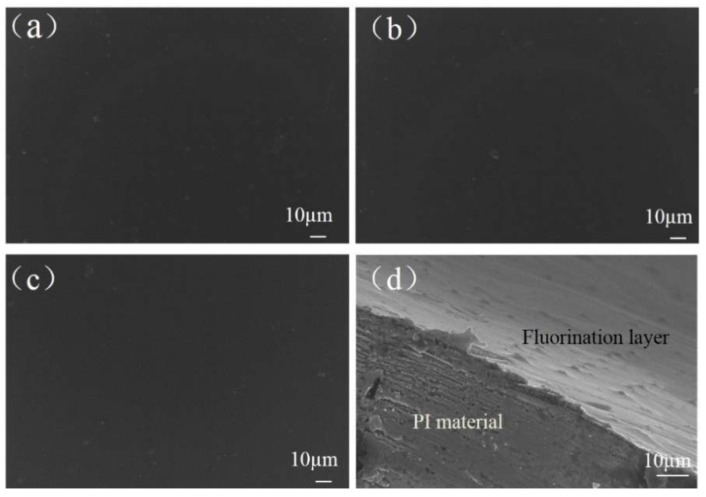
Surface morphologies of the PI dielectrics with fluorination time of (**a**) 0 min, (**b**) 30 min and (**c**) 60 min, cross-section morphology of (**d**) 60 min fluorinated dielectric.

Figure 11 shows surface morphologies of the PI dielectrics before plasma discharge aging. As can be seen, there was no significant difference between surfaces of the un-fluorinated PI dielectric and the fluorinated PI dielectric; all of them had smooth surfaces without potholes, except some occasionally distributed micro size dust particles. Cross-section image of the 60 min fluorinated PI dielectric is given in Figure 11d. It can be seen that a fluorinated layer was formed after fluorination, but the matrix of the dielectric is not affected.

**Figure 12:**
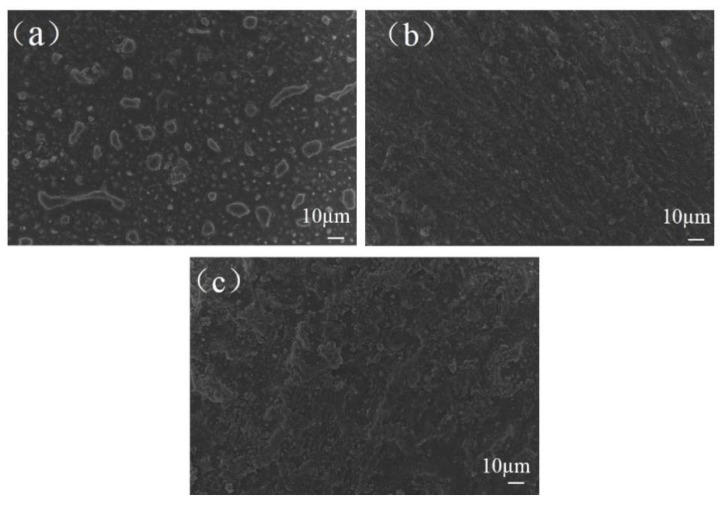
Surface morphologies of the PI dielectrics area after discharge aging: un-fluorinated PI (**a**), 30 min fluorinated PI (**b**), 60 min fluorinated PI (**c**).

Figure 12 shows surface morphologies of the PI dielectrics after 10 h of plasma processing. All the images were taken in the plasma discharge region around the edge of the exposed electrode. It can be clearly observed that the plasma processing caused distinct changes of surface morphologies. As shown in Figure 12a, the surface of the un-fluorinated PI was severely etched and damaged, many irregular small gibbosities remained on the surface, around which there were lots of bubbles. These gibbosities and bubbles were the PI material and the underlying Si-based adhesive, respectively. The isolated gibbosity-like morphology can be ascribed to the space among the randomly distributed micro-discharge, where the active species are generated along the filamentary channel and spatially uneven distributed in the discharge regime [40]. By contrast, Figure 12b,c show that surface morphologies of 30 and 60 min fluorinated PI dielectrics were more integrated, and the surface roughness was increased after discharge aging. However, no obvious differences in the morphologies can be found between the two surfaces.

XPS analysis was further conducted to investigate changes in the surface chemical composition of un-fluorinated PI and fluorinated PI, before and after plasma processing. As shown in Table 1, for the un-fluorinated PI after 10 h of plasma processing, the concentrations of both carbon and nitrogen elements decreased sharply, while the concentration of oxygen element increased remarkably from 17.81% to 38.72%. Besides, a new element (Si) was detected and accounted for 28.57% of chemical composition; this was due to the fact that long plasma processing caused the exposure of the underlying Si-based adhesive. Compared to un-fluorinated PI, F element was detected on the surfaces of fluorinated PI. When the fluorination time increased from 30 to 60 min, there was no distinct difference between surface chemical composition of 30 min (2#-0 h) and 60 min (3#-0 h) fluorinated PI dielectrics. However, after 10 h of plasma processing, it can be seen that F element concentration of 60 min fluorinated PI was about 2.3 times as the 30 min fluorinated one.

**Figure 13:**
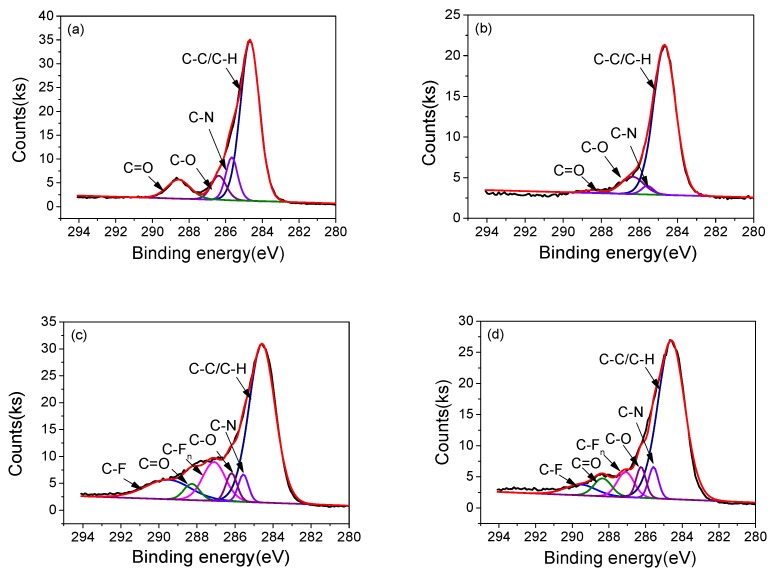

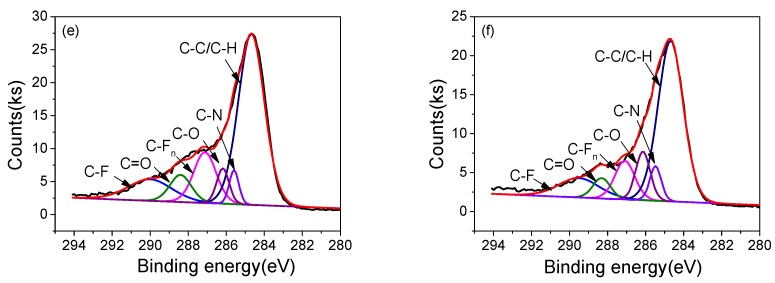
High resolution XPS spectra of C1s peak of (**a**) unaged and (**b**) 10 h aged un-fluorinated PI; (**c**) unaged and (**d**) 10 h aged 30 min fluorinated PI; (**e**) unaged and (**f**) 10 h aged 60 min fluorinated PI.

In order to examine the changes of functional carbon groups on the surfaces, deconvolution analysis of the C1s peaks were executed; the results are shown in Figure 13 and Table 2. It is noted that C1s spectra of un-fluorinated PI had four peaks, namely, C–C/C–H, C–N, C–O, and C–N, which respectively corresponded to the binding energies of 284.7, 285.6, 286.3, and 288.6 eV [24,25]. In addition to the above four peaks, the C1s spectra of surface fluorinated PI incorporated two new peaks, at the binding energies of 287.1 and 289.5 eV, which corresponded to the C–F_n_ and C–F peaks [26], respectively.

For un-fluorinated PI, the concentrations of C–C/C–H, C–N, C–O, and C=O groups were 69.65%, 11.87%, 8.3% and 10.17%, respectively. After 10 h of plasma processing, the C–C/C–H group increased sharply to 85.78% while the concentration of C–N group decreased significantly to 2.64%. Meanwhile, the concentration of C=O also showed a sharp decreasing tendency. These changes can be explained by the reactive and heat effect of DBD micro-discharge region on the PI surface. For the fluorinated PI before plasma processing, when fluorination time was increased from 0 to 30 and 60 min, the concentration of C–C/C–H group decreased sharply from 69.65% to 61.28% and 56.44%, while concentration of C–F group increased to 13.06% and 13.49%, and concentration of C–F_n_ group increased to 13.05% and 13.01%. This result revealed that the C–C/C–H bonds can be easily broken during plasma processing. Besides, there was no significant concentration variation of fluorine-containing groups when fluorination time was increased from 30 to 60 min, which agreed well with the surface elements composition. After 10 h of plasma processing, the fluorine-containing group concentration of the 60 min fluorinated PI (~22.15%) was much higher than that of the 30 min fluorinated PI (~12.84%).

## 4. Discussion

The dielectric lifetime of multi-layered PI-based SDBD device in atmospheric air has an obvious extension through dielectric surface fluorination modification. From surface properties of dielectrics, it can be inferred that two main reasons result in longer dielectric lifetime.

Firstly, the physical effect works, which was reflected in lower surface temperature and increased surface roughness. In aspect of surface temperature distribution, it can be seen from Figure 7 that compared to un-fluorinated PI, the existence of the surface fluorination layer contributed to lower surface temperature, meaning that more effective heat dissipation can be obtained when using fluorinated PI dielectrics. In another aspect of surface morphology, due to an etching effect of plasma discharge, surface roughness increased for the fluorinated PI as shown in Figure 12b,c while the top layer was almost removed for the un-fluorinated multi-layered PI; this indicates that the fluorination layer had positive effects on plasma etching inhibition. In addition, during the plasma process, rougher surface caused a variation of electron movement trajectory and the increase of diffuse reflection [41]. The travel distance of secondary electrons then decreased, that is to say they cannot gain sufficient energy from the electrical field, subsequently, collision energy between the electrons and the dielectric surface was also weakening.

Secondly, the chemical effect works, which was reflected in the graft of fluorine groups. The fluorine groups were introduced into the PI surface after fluorination and led to changes of the surface properties. On the one hand, as shown in Table 1, after 10 h of plasma discharge, the F element concentration of 60 min fluorinated PI was still 2.3 times higher than that of the 30 min fluorinated PI. Because of the electronegativity of F element, electrons were significantly adsorbed by F atoms. Consequently, the collisions between the electrons and dielectric surface were restrained. The higher concentration of the F element, more obvious was the restrain effect. On the other hand, it is well known that direct fluorination of polymer results in the disruption of C–H bond and a formation of C–F and C–F_n_ groups, as C–F bond is the strongest single bond in organic chemistry and has bond dissociation energy of 544 kJ/mol [42], much higher than that of the C–H bond (414 kJ/mol) [43]. Thus, C–F bond can resist the bombardment of the active species more effectively than the other bonds. As shown in Table 2, after 10 h of plasma discharge, the C–F group concentration of the 60 min fluorinated PI remained at 11.21%, about two times higher than that of the 30 min fluorinated PI.

## 5. Conclusions

In this work, discharge, plasma and dielectric material characteristics of fluorinated PI based SDBD plasma device are studied. During the discharge aging process, the changes of plasma parameters, including gas temperature and electron temperature, are lower for the fluorinated PI based device than the un-fluorinated case, showing that more stable discharge can be obtained when using fluorinated PI as a barrier dielectric. In addition, the dielectric lifetime extends with the fluorination time. The lifetime of the 60 min fluorinated PI-based plasma device is about three times longer than that of the un-fluorinated case at 10 kV applied peak to peak voltage and 6 kHz frequency, which can be explained by surface temperature, roughness, and functional groups changes on the surface of PI. Firstly, after discharge aging, the distinct surface temperature increase of the un-fluorinated PI illustrates that it accumulates more heat energy, which has adverse effects on long-time operation, while no significant temperature variation can be observed on the surface of the 60 min fluorinated PI. Secondly, the formed rougher surface from fluorinated PI leads to reduction of collision energy between the secondary electrons and dielectric surface in the discharge region. Finally, XPS results show that the fluorine-containing groups are incorporated into the surface of the PI dielectrics; since their bond energy is much higher than C–C/C–H, material degradation effectively slows down during the plasma processing. These results suggest that surface fluorination is a suitable modification method of the dielectric barrier material for application in long lifetime SDBD plasma devices.

## Figures and Tables

**Table 1:** Relative chemical composition as determined by XPS before and after plasma processing.

Samples	Chemical composition (at %)				
	C	N	O	F	Si
1#-0h	78.32	3.87	17.81	—	—
1#-10h	31.96	0.75	38.72	—	28.57
2#-0h	54.28	3.7	14.94	27.08	—
2#-10h	63.14	6.41	23.55	6.9	—
3#-0h	52.14	3.88	16.91	27.07	—
3#-10h	51.09	7.54	25.40	15.97	—

**Table 2:** Relative surface chemical bond content of the PI surfaces before and after plasma aging.

Samples	Relative area of different chemical bonds (%)					
	C 1s possible groups					
	C–C/C–H	C–N	C–O	C=O	C–F_n_	C–F
1#-0h	69.65	11.87	8.3	10.17	—	—
1#-10h	85.78	2.64	10.26	1.32	—	—
2#-0h	61.28	4.11	4.69	3.80	13.06	13.05
2#-10h	71.58	5.15	5.09	2.76	7.21	5.63
3#-0h	56.44	4.10	5.54	7.19	13.49	13.01
3#-10h	57.21	5.64	9.94	5.06	10.94	11.21
